# Comparative analysis of trends in the burden of pancreatitis in China and worldwide, 1990–2021

**DOI:** 10.3389/fpubh.2025.1616215

**Published:** 2025-12-05

**Authors:** Jianwang Cai, Zhengwu Jiang, Zhaoming Yang, Zhijian Zhao, Jian Wan, Xun Chen

**Affiliations:** 1 Second Department of Hepatobiliary Pancreatic and Splenic Surgery, Medical Center of Digestive Disease, Zhuzhou Hospital Affiliated to Xiangya School of Medicine, Central South University, Zhuzhou, Hunan, China; 2 Jishou University Zhuzhou Clinical College, Medical College, Jishou University, Zhuzhou, Hunan, China

**Keywords:** pancreatitis, trend, incidence, mortality, prevalence, disability-adjusted life years

## Abstract

**Background:**

Amidst global demographic aging patterns, understanding divergent trajectories of pancreatitis burden is crucial for health equity. This study compares China’s pancreatitis burden (1990–2021) against global aggregates, focusing on age-sex stratification within aging populations.

**Methods:**

Leveraging Global Burden of Disease 2021 datasets, we analyzed age-standardized rates (ASR) for incidence (ASIR), prevalence (ASPR), mortality (ASMR), and disability-adjusted life years (DALYs/ASDR). Joinpoint regression quantified temporal trends via average annual percentage changes (AAPC). Statistical significance was determined through 95% confidence intervals (CIs). GBD standardization protocols ensured comparability of sex-age stratified analyses.

**Results:**

From 1990 to 2021, China witnessed significant declines in pancreatitis burden. The age-standardized rates per 100,000 population fell as follows: incidence (ASIR) from 35.35 to 23.95; prevalence (ASPR) from 35.33 to 24.15; mortality (ASMR) from 0.98 to 0.64; and DALYs (ASDR) from 29.77 to 18.27. The corresponding average annual percentage changes (AAPCs: ASIR −1.34%; ASPR −1.25%; ASMR −1.40%; ASDR −1.57%) were substantially greater than the global averages (ASIR −0.44%; ASPR −0.99%; ASMR −0.47%; ASDR −0.53%). Age and sex disparities were clear, with males having higher rates below age 65, and females bearing a greater burden thereafter.

**Conclusion:**

This study highlights a marked decline in China’s pancreatitis burden, linked to healthcare advances, despite persistent disparities in older populations. Moving beyond isolated descriptions of trends, our study introduces a direct comparative analysis that quantifies China’s steeper decline in pancreatitis burden relative to the global average. This provides a nuanced, quantitative benchmark for evaluating the effectiveness of national health policies. These findings call for equitable policies and future work on region-specific risks and scalable interventions.

## 1 Introduction

Pancreatitis, a prevalent digestive system disorder with diverse etiologies and complex clinical manifestations, continues to impose a growing global disease burden (1). For instance, acute pancreatitis represents a leading cause of hospitalization for acute abdominal conditions, with mortality rates exceeding 20% in severe cases. Chronic pancreatitis, meanwhile, severely compromises patient quality of life due to recurrent complications such as pancreatic pseudocysts and diabetes (2).

In recent decades, shifting global demographics—including population aging, lifestyle modifications, and dietary pattern transitions—have contributed to heterogeneous trends in pancreatitis incidence and burden across regions (3, 4). Particularly in China, the challenges posed by an aging population demand adaptive public health strategies. As highlighted by Chen et al. (5), integrating chronic disease management within the framework of healthy aging is critical to mitigating the health burden of conditions such as pancreatitis. Nevertheless, marked disparities persist in burden characteristics and their drivers among different geographic, sex, and age groups, highlighting the urgent need for more comprehensive and precise epidemiological investigations.

Existing evidence indicates substantial global disparities in pancreatitis incidence and mortality across nations, potentially attributable to inequitable distribution of health resources, diagnostic capacity limitations, and differential exposure to major risk factors such as biliary diseases, alcohol consumption, and hyperlipidemia (6, 7). In high-income countries, medical costs and socioeconomic burdens associated with pancreatitis are notably elevated, whereas management and prognosis remain critically constrained in resource-limited regions. Sex and age further shape pancreatitis burden, with males typically exhibiting higher acute pancreatitis incidence (8), while females in older populations demonstrate disproportionately elevated mortality rates and disability-adjusted life years (DALYs). These divergences underscore the heterogeneous nature of pancreatitis burden and the imperative for sex- and age-sensitive health policies. In China, the world’s most populous nation, pancreatitis has emerged as a pressing public health challenge. A study by Yu et al. revealed a marked increase in pancreatitis burden among Chinese older adults populations over the past three decades, particularly in those aged ≥65 years. Projections by Wen et al. suggest continued escalation of pancreatitis burden both globally and in China over the next decade, with widening health inequities anticipated (3, 4). Collectively, these findings highlight the necessity for in-depth epidemiological analyses of disease trends and driving mechanisms through multidisciplinary collaboration and global data integration (7).

This study leverages data from the Global Burden of Disease (GBD) Study (1990–2021) to systematically assess temporal trends in pancreatitis burden in China, with comparative evaluations against global benchmarks. By analyzing variation patterns across sex, age groups, and temporal dimensions, we delineate the evolving burden of pancreatitis in China over the past three decades. These findings aim to inform evidence-based decision-making for policymakers and clinicians, support optimization of prevention and management strategies, and guide targeted efforts to mitigate future disease burden.

Although the temporal trends of pancreatitis have been described in various settings, a systematic and quantitative analysis that directly benchmarks China’s trajectory against the global average—using uniform GBD metrics and analytical frameworks—has been lacking. This study fills this critical gap by providing a direct, head-to-head comparative analysis, which is essential to delineate China’s unique epidemiological trajectory and to derive actionable insights for global health equity.

## 2 Methods

### 2.1 Overview

This study systematically analyzes the temporal trends of pancreatitis disease burden in China from 1990 to 2021, encompassing incidence, prevalence, mortality, and disability-adjusted life years (DALYs). Building upon data from the Global Burden of Disease (GBD) Study, we conduct an objective assessment of epidemiological patterns across sex and age-specific populations. Through a comprehensive analysis of these disease burden indicators, we aim to elucidate critical epidemiological characteristics of pancreatitis, providing scientific evidence to guide the development of targeted prevention and control policies.

### 2.2 Data sources

The data for this study were derived from the Global Burden of Diseases, Injuries, and Risk Factors Study (GBD) 2021 database, encompassing pancreatitis-related disease burden metrics — including incidence, prevalence, mortality, and disability-adjusted life years (DALYs) — across 204 countries and territories from 1990 to 2021. The GBD database employs validated modeling tools such as DisMod-MR (for estimating disease incidence and prevalence) and CODEm (for analyzing cause-specific mortality patterns) (9, 10). Data were retrieved from the Global Health Data Exchange (GHDx) platform, comprising publicly available, anonymized datasets that strictly adhere to ethical guidelines, thereby ensuring methodological transparency and regulatory compliance (11).

### 2.3 Analysis scope

This study focused on pancreatitis-related disease burden in China and globally, stratified by sex and age groups between 1990 and 2021. Core disease burden metrics included age-standardized incidence rate (ASIR), age-standardized prevalence rate (ASPR), age-standardized mortality rate (ASMR), and age-standardized disability-adjusted life years rate (ASDR). Additionally, crude incidence rate (CIR), crude prevalence rate (CPR), crude mortality rate (CMR), and crude DALY rate (CDR) were analyzed. These combined indicators provide a comprehensive evaluation of pancreatitis burden trends across populations, enabling granular assessments of temporal and demographic variations (10, 11).

### 2.4 Joinpoint regression analysis

To assess temporal trends and inflection points in disease burden, this study employed Joinpoint regression methodology (12). The Joinpoint model identifies significant trend transitions and computes the average annual percentage change (AAPC) using the equation: *ln*(*y*) = *α* + *βx* + *ε*, where *y* represents disease burden metrics, *x* denotes calendar year, and *β* is the regression coefficient. The AAPC is calculated as *AAPC = 100 × (exp(β) − 1)*, with its statistical significance determined by 95% confidence intervals (CIs). Trends were considered statistically significant if the 95% CI of the AAPC excluded the null value (i.e., 0) (13). All analyses were implemented using Joinpoint software (National Cancer Institute, Version 5.0.2).

## 3 Results

### 3.1 Incidence of pancreatitis in China and globally

Regarding incidence, the total number of cases in China increased from 361,363 (95% CI: 297,459–429,207) in 1990 to 440,292 (95% CI: 376,057–514,656) in 2021. However, the age-standardized incidence rate (ASIR) declined from 35.35 per 100,000 (95% CI: 29.77–41.42) in 1990 to 23.95 per 100,000 (95% CI, 18.62–29.89) in 2021, with an average annual percentage change (AAPC) of −1.34% (95% CI, −1.45 to −1.23). This suggests that while the absolute number of cases rose, the ASIR decreased due to demographic aging and public health interventions. Globally, the total incidence increased from 1,728,141 cases (95% CI, 1,495,096–1,995,752) in 1990 to 2,747,368 (95% CI, 2,413,878–3,133,076) in 2021. Similarly, the global ASIR showed a declining trend, falling from 37.62 per 100,000 (95% CI, 32.57–43.46) to 32.81 per 100,000 (95% CI, 28.85–37.38) during the same period (AAPC: −0.44%; 95% CI: −0.47 to −0.41) (Table 1). These findings indicate a gradual stabilization of pancreatitis incidence worldwide, while China’s healthcare reforms and targeted policies over the past decades have contributed significantly to its accelerated reduction in age-standardized rates (Table 1).

**Table 1:** All-age cases and age-standardized incidence rate, prevalence rate, mortality rate, DALYs rate of pancreatitis in China and worldwide for 1990 and 2021, with corresponding average annual percentage changes (AAPC).

Location	Measure	1990 all-ages cases (95% UI)	2021 all-ages cases (95% UI)	1990 ASR (95% UI) (per 100,000)	2021 ASR (95% UI) (per 100,000)	AAPC (95% CI) (%)
China	Incidence	361,363 (297459–429,207)	440,292 (376057–514,656)	35.35 (29.77–41.42)	23.95 (18.62–29.89)	−1.34 (−1.45 – −1.23)
	Prevalence	352,010 (238431–508,156)	488,128 (344036–692,780)	35.33 (23.78–50.99)	24.15 (17.33–33.29)	−1.25 (−1.37 – −1.12)
	Deaths	7,831 (6124–9,648)	12,156 (9212–15,725)	0.98 (0.77–1.24)	0.64 (0.49–0.81)	−1.40 (−1.56 – −1.24)
	DALYs	294,678 (238378–361,020)	347,015 (271175–444,968)	29.77 (23.87–36.36)	18.27 (14.35–23.42)	−1.57 (−1.76 – −1.39)
Global	Incidence	1,728,141(1495096–1,995,752)	2,747,368 (2413878–3,133,076)	37.62 (32.57–43.46)	32.81 (28.85–37.38)	−0.44 (−0.47 – −0.41)
	Prevalence	4,148,305 (3042449–5,639,570)	5,900,340 (4376043–7,838,798)	93.74 (68.31–127.08)	68.99 (51.26–91.29)	−0.99 (−1.03 – −0.95)
	Deaths	68,490 (60748–78,272)	122,416 (109848–141,362)	1.69 (1.50–1.92)	1.45 (1.30–1.67)	−0.47 (−0.64 – −0.29)
	DALYs	2,583,402 (2265738–2,985,509)	4,101,154 (3647631–4,684,283)	57.39 (50.34–66.07)	48.43 (43.07–55.35)	−0.53 (−0.64 – −0.41)

### 3.2 Prevalence of pancreatitis in China and globally

In China, the total number of prevalent cases increased from 352,010 (95% CI: 238,431–508,156) in 1990 to 488,128 (95% CI: 344,036–692,780) in 2021. However, the age-standardized prevalence rate (ASPR) decreased from 35.33 per 100,000 (95% CI: 23.78–50.99) in 1990 to 24.15 per 100,000 (95% CI: 17.33–33.29) in 2021, with an average annual percentage change (AAPC) of −1.25% (95% CI: −1.37 to −1.12). These trends indicate that despite the rising absolute prevalence, improved disease prevention and management strategies have effectively reduced the age-standardized burden. Globally, the total prevalent cases rose from 4,148,305 (95% CI: 3,042,449–5,639,570) in 1990 to 5,900,340 (95% CI: 4,376,043–7,838,798) in 2021. The global ASPR declined from 93.74 per 100,000 to 68.99 per 100,000 during this period. Notably, the increasing crude prevalence in certain low-income countries and regions may reflect disparities in healthcare access, socioeconomic challenges, and delayed diagnosis or treatment (Table 1).

### 3.3 Mortality of pancreatitis in China and globally

In China, the total number of deaths increased from 7,831 (95% CI: 6,124–9,648) in 1990 to 12,156 (95% CI: 9,212–15,725) in 2021. However, the age-standardized mortality rate (ASMR) decreased from 0.98 per 100,000 (95% CI: 0.77–1.24) in 1990 to 0.64 per 100,000 (95% CI: 0.49–0.81) in 2021, with an average annual percentage change (AAPC) of −1.40% (95% CI: −1.56 to −1.24). This demonstrates that population growth and aging drove the rise in absolute deaths, while advancements in medical care contributed to reduced mortality rates. Globally, similar trends were observed: total deaths from pancreatitis increased, yet the ASMR declined consistently (Table 1). These patterns are likely attributable to improved early diagnosis, optimized therapeutic approaches, and enhanced healthcare accessibility in many regions (Table 1).

### 3.4 Disease burden of pancreatitis measured by disability-adjusted life years (DALYs)

In China, the total number of pancreatitis-related DALYs increased from 294,678 (95% CI: 238,378–361,020) in 1990 to 347,015 (95% CI: 271,175–444,968) in 2021. However, the age-standardized DALYs rate (ASDR) exhibited a significant decline, dropping from 29.77 per 100,000 (95% CI: 23.87–36.36) in 1990 to 18.27 per 100,000 (95% CI: 14.35–23.42) in 2021, with an average annual percentage change (AAPC) of −1.57% (95% CI: −1.76 to −1.39). Globally, a similar downward trend in ASDR was observed, though the magnitude of reduction was smaller (AAPC −0.53, 95% CI: −0.64 to −0.41) compared to China (Table 1). Despite rising total DALYs worldwide—largely attributable to population growth and aging—the decline in ASDR highlights the substantial impact of strengthened public health interventions, healthcare system advancements, and optimized disease management strategies in mitigating the disease burden (Table 1).

### 3.5 Joinpoint regression analysis of the burden of pancreatitis in China and worldwide

**Figure 1:**
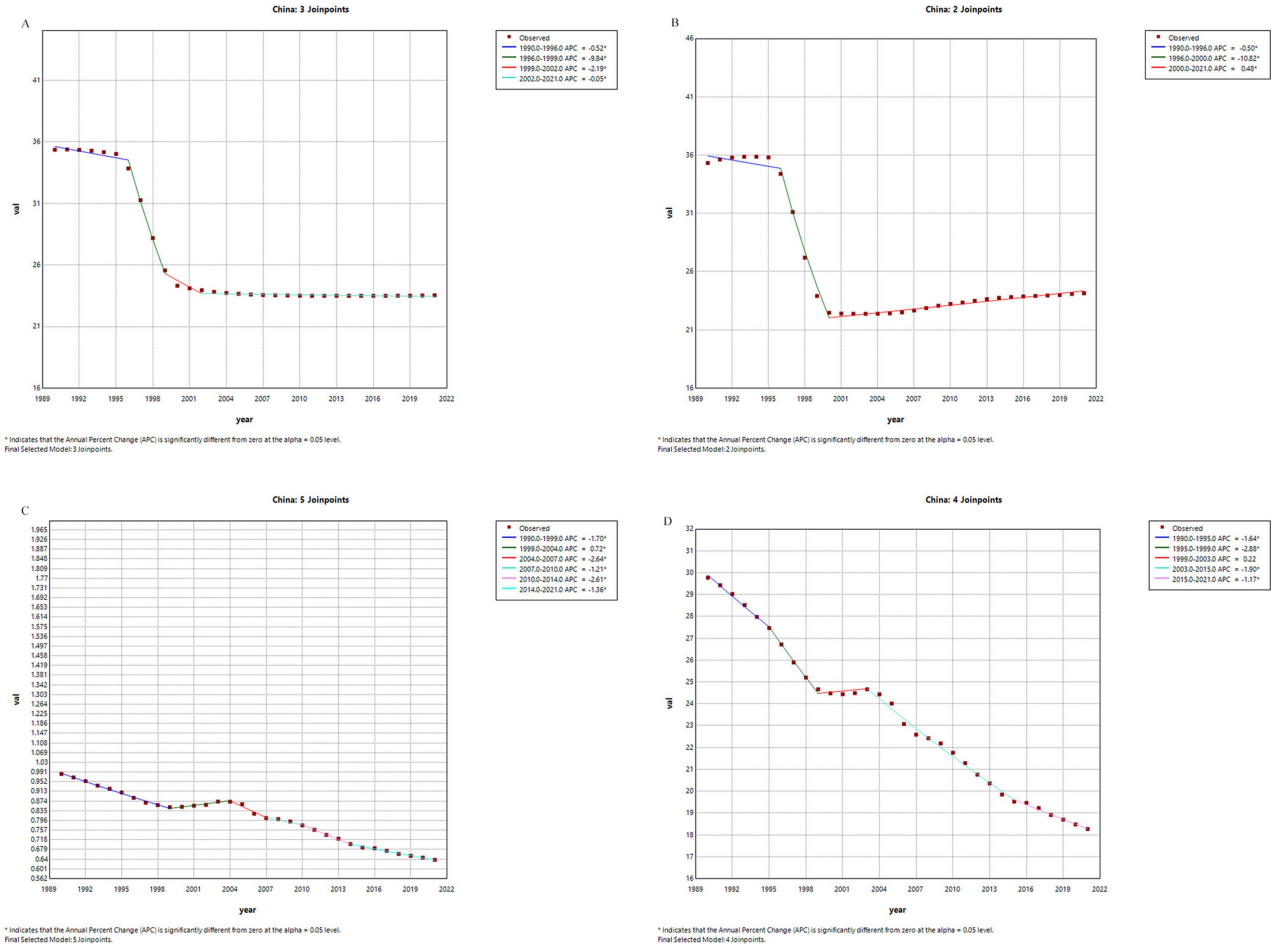
Trends in APC of pancreatitis burden metrics in China, 1990–2021. **(A)** ASIR. **(B)** ASPR. **(C)** ASMR. **(D)** ASDR. *p* < 0.05 (*); Data from GBD 2021.

**Figure 2:**
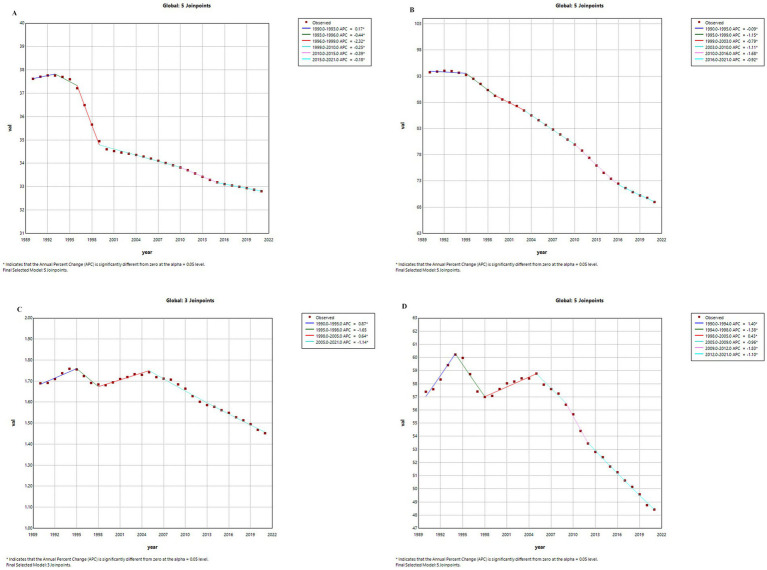
Trends in APC of pancreatitis burden metrics worldwide, 1990–2021. **(A)** ASIR. **(B)** ASPR. **(C)** ASMR. **(D)** ASDR. *p* < 0.05 (*); Data from GBD 2021.

Figures 1, 2 present the joinpoint regression analysis results of age-standardized incidence rate (ASIR), prevalence rate (ASPR), mortality rate (ASMR), and disability-adjusted life years rate (ASDR) for pancreatitis in China and globally from 1990 to 2021. China: From 1990 to 1996, both ASIR (annual percentage change, APC = −0.52, *p* < 0.05) and ASPR (APC = −0.5, *p* < 0.05) showed significant declines. Over the entire study period, ASMR and ASDR also exhibited sustained downward trends. Global Trends: ASIR and ASPR similarly declined significantly worldwide. However, the APC for global ASMR and ASDR fluctuated between 1990 and 2005, followed by a significant reduction in annual decline rates since 2005. Key Conclusion: All indicators in China demonstrated varying degrees of decline, with more pronounced reductions compared to global trends.

### 3.6 Trend in the burden of pancreatitis in China and globally

**Figure 3:**
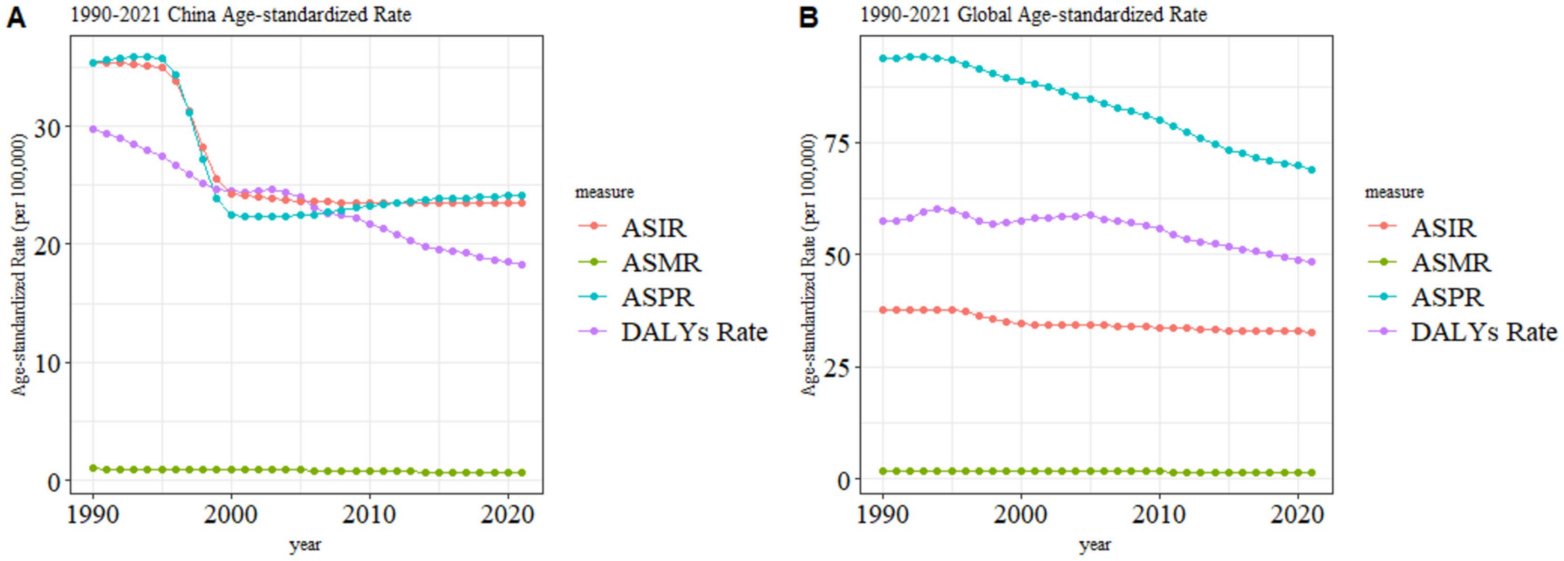
Comparison of pancreatitis burden trends between China and the global population, 1990–2021. **(A)** China Age-standardized Rate. **(B)** Global Age-standardized Rate. *p* < 0.05 (*); Data from GBD 2021.

Figure 3 compares the ASIR, ASPR, ASMR, and ASDR of pancreatitis between China (A) and the global population (B) from 1990 to 2021. In China, the ASIR significantly declined between 1990 and 2000, stabilizing at approximately 20 per 100,000 population thereafter. The ASPR decreased from 1990 to 2000 but exhibited a slight upward trend post-2000. Both ASMR and ASDR demonstrated continuous declines throughout the period, though the ASMR reduction was not statistically significant. Globally, ASMR remained stable, while ASDR fluctuated between 1990 and 2005, followed by a steady decline after 2005. A significant downward trend was observed in ASPR, whereas ASIR experienced a modest decline.

### 3.7 Geographic disparities in pancreatitis burden

**Figure 4:**
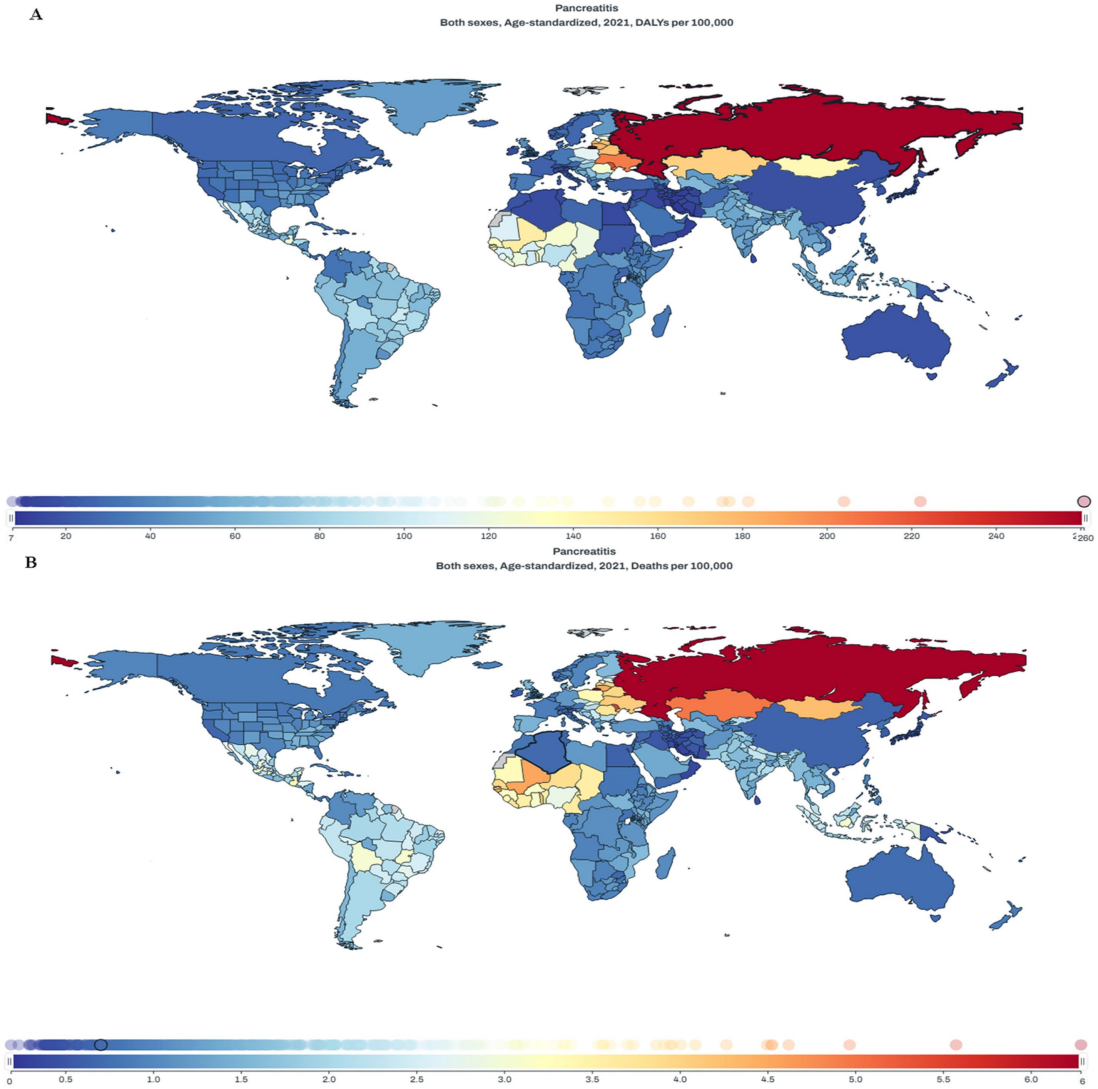
Global distribution of the age-standardized burden of pancreatitis in 2021. **(A)** Age-standardized disability-adjusted life years rate (ASDR). **(B)** Age-standardized mortality rate (ASMR). Data were obtained from the GBD 2021 study.

To visually depict the spatial heterogeneity of the pancreatitis burden, we present global maps of the age-standardized disability-adjusted life years rate (ASDR) and mortality rate (ASMR) for the year 2021 (Figure 4). The data reveal substantial spatial inequalities on a global scale. In terms of overall burden (ASDR, Figure 4A), Eastern Europe bore the heaviest load, with a rate of 260.76 per 100,000. This stands in stark contrast to the much lower burdens observed in North America (27.52 per 100,000) and Australia (19.86 per 100,000). China’s ASDR was 18.27 per 100,000, positioning it in the low-to-middle range globally. A similarly pronounced disparity was observed for fatal outcomes (ASMR, Figure 4B). Eastern Europe (e.g., Russia, 6.28 per 100,000) and parts of Central Asia (e.g., Kazakhstan, 4.97 per 100,000) exhibited the highest mortality rates. Conversely, the lowest rates were found in Western Europe (e.g., France, 0.90 per 100,000), North America (0.85 per 100,000), and Oceania (0.72 per 100,000). China’s ASMR was 0.64 per 100,000, which is comparable to, or even lower than, the rates in several high-income nations. In summary, the burden of pancreatitis in 2021 demonstrated extreme global inequality, with a more than 10-fold difference in ASDR and ASMR between the highest and lowest burden regions. China occupies a relatively favorable position for both metrics.

### 3.8 Age-specific burden of pancreatitis in China: 1990 vs. 2021

**Figure 5:**
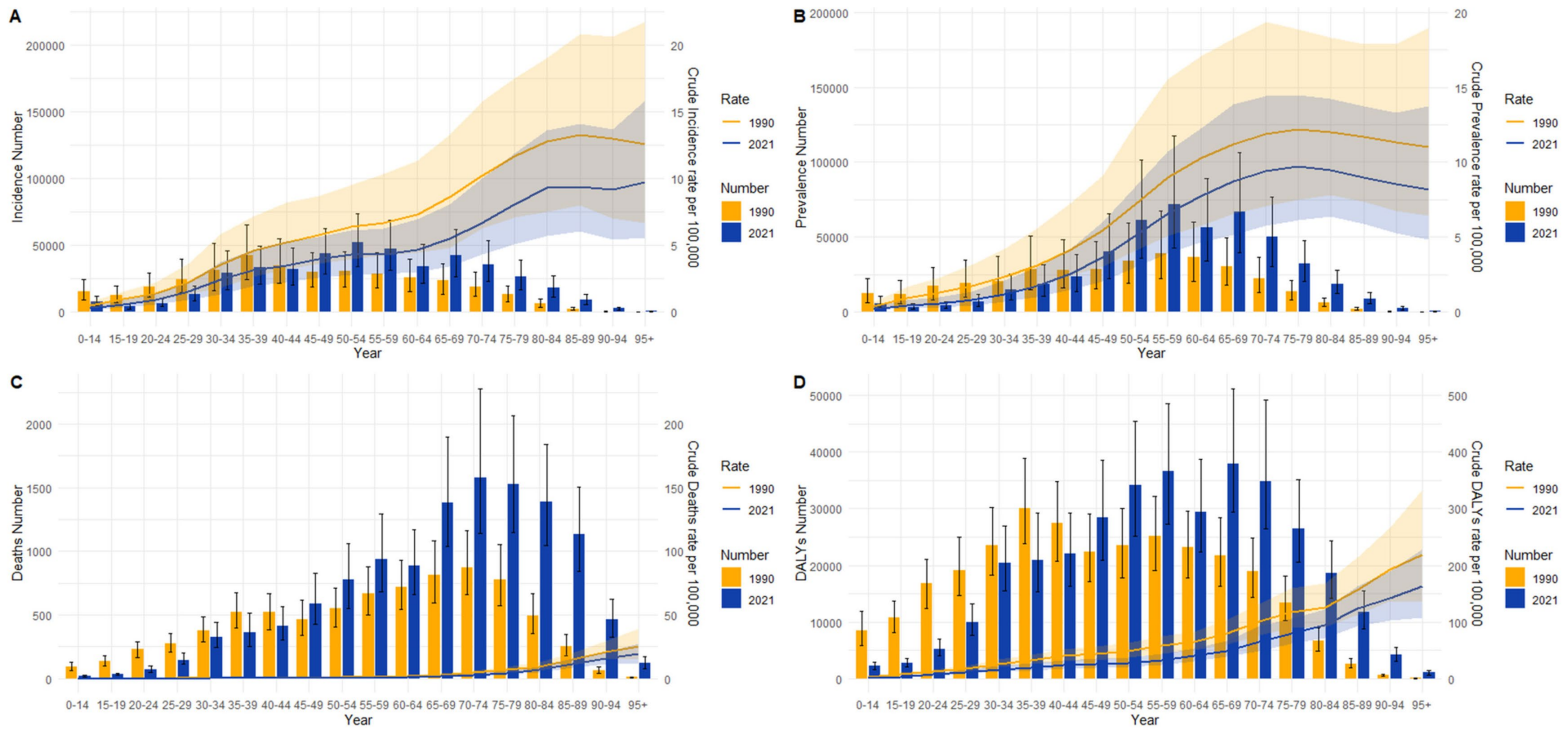
Comparison of pancreatitis burden by age group in China (1990 vs 2021): Counts and crude rates of incidence, prevalence, mortality, and disability-adjusted life years (DALYs). **(A)** Incident case counts and crude incidence rate (CIR). **(B)** Prevalent case counts and crude prevalence rate (CPR). **(C)** Death counts and crude mortality rate (CMR). **(D)** DALYs counts and crude DALYs rate (CDR). *B*ar charts represent counts; line charts indicate crude rates.

Figure 5 displays the age-specific distribution of crude incidence rate (CIR), prevalence rate (CPR), mortality rate (CMR), and crude disability-adjusted life years rate (CDR) for pancreatitis in China in 1990 and 2021. Figure 5A shows that CIR increased significantly with advancing age in both years. Figure 5B demonstrates a similar trend for CPR, with prevalence rates rising substantially across all age groups, particularly among those aged ≥65 years. The number of prevalent cases peaked in the 55–59 age group. This pattern aligns with global trends observed in CIR and CPR (Supplementary Figure 1). Figure 5C highlights a marked increase in CMR and deaths among older populations, with the highest mortality counts occurring in the 70–74 age group. Figure 5D indicates significantly elevated CDR values and case numbers in older adults populations. Comparative analysis of prevalence counts revealed substantial increases in 2021 relative to 1990, most pronounced in the 65–69 age group. All panels consistently demonstrate the disproportionate impact of pancreatitis burden on older populations, accompanied by higher incident case counts in 2021 compared to 1990. Similar age-specific patterns were documented globally (Supplementary Figure 1).

### 3.9 Gender disparities in pancreatitis burden across age groups in China

**Figure 6:**
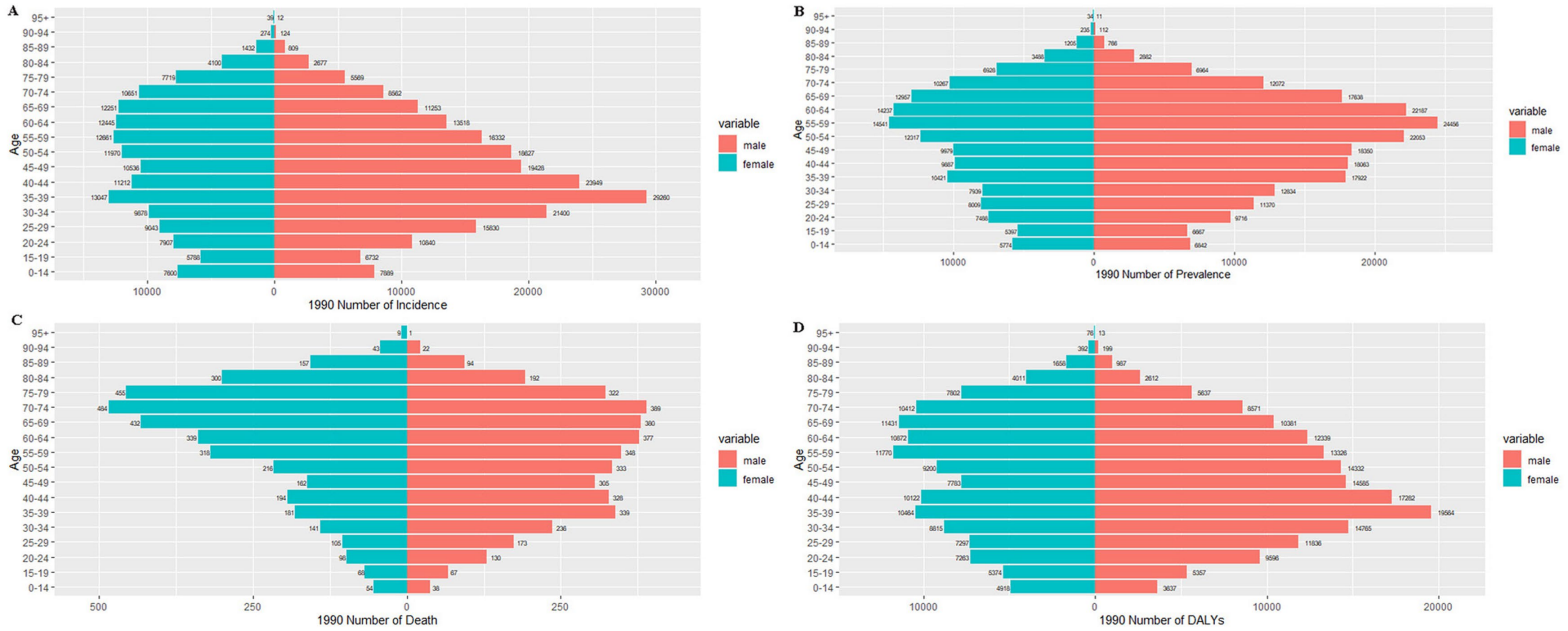
Sex-stratified comparison of pancreatitis burden by age group in China, 1990: incidence, prevalence, mortality, and disability-adjusted life years (DALYs). **(A)** Incidence rate; **(B)** prevalence rate; **(C)** mortality rate; **(D)** DALYs.

**Figure 7:**
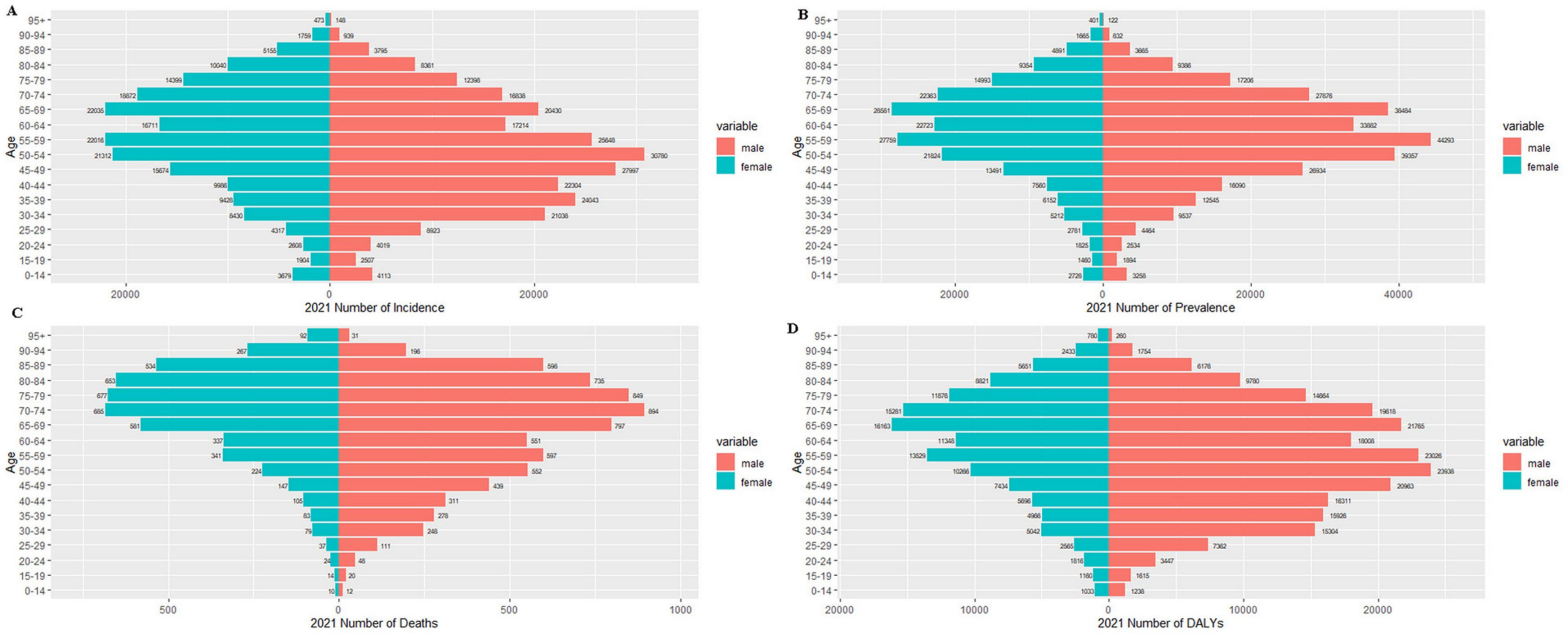
Sex-stratified comparison of pancreatitis burden by age group in China, 2021: incidence, prevalence, mortality, and disability-adjusted life years (DALYs). **(A)** Incidence rate; **(B)** prevalence rate; **(C)** mortality rate; **(D)** DALYs.

Figures 6, 7 present the age-stratified incidence, prevalence, mortality, and disability-adjusted life years (DALYs) of pancreatitis in males and females in China for 1990 and 2021.

An analysis of incidence rates revealed that the highest number of new acute pancreatitis (AP) cases in 1990 was observed in the 35–39 age group, with a gradual decline in older cohorts, mirroring global patterns (Supplementary Figure 2). While males exhibited consistently higher incidence rates than females in most age groups, this trend reversed in individuals over 65 years, where female incidence surpassed that of males (Figure 6A). By 2021, the incidence trajectory remained similar, with males continuing to dominate in younger and middle-aged groups. Notably, the older adults population (≥65 years) showed a modest increase in total case counts compared to 1990 levels (Figure 7A).

The pattern of disease prevalence in 1990 demonstrated an age-dependent progression pattern, with case counts rising from younger cohorts, peaking in the 55–59 age group, then gradually decreasing in advanced ages. While males maintained higher prevalence rates across all age groups, this pattern showed exceptions in octogenarians and beyond (≥80 years), where females exhibited marginally greater case numbers (Figure 5B). By 2021, prevalence trends largely retained this configuration, with males continuing to predominate in younger (<60 years) and middle-aged (60–74 years) populations. Notably, the female population demonstrated increased case shares in advanced aging subgroups, particularly surpassing male prevalence in the ≥80 age bracket (Figure 7B). Globally, while similar epidemiological patterns were observed for acute pancreatitis (AP) prevalence, cross-national comparisons reveal a more pronounced female predominance in older adults (≥65 years) than seen in China’s context (Supplementary Figure 2).

Distinct gender-specific trends in pancreatitis mortality were observed. In 1990, mortality rates for both sexes exhibited age-dependent escalation, peaking in the 70–74 age cohort before declining in older populations. Males demonstrated higher death counts in younger (<65 years) and middle-aged (65–74 years) groups, while females showed marginally elevated mortality among the older adults (≥75 years), aligning with global epidemiological patterns (Figure 6C and Supplementary Figure 2). By 2021, mortality profiles displayed nuanced shifts: younger cohorts (<50 years) experienced reduced fatalities compared to 1990 baselines, yet the 70–74 age group remained the peak mortality stratum. A pronounced female predominance emerged in extreme aging populations (≥90 years), contrasting with persistently higher male mortality in younger demographics (Figure 6C).

#### 3.9.1 DALY dynamics across the lifespan

In 1990, sex-specific DALY burdens revealed stark epidemiological divides: males exhibited elevated disability-adjusted life year (DALY) burdens in younger (<45 years) and middle-aged (45–64 years) cohorts, whereas females carried disproportionate burdens in advanced aging populations (≥65 years). Sex-disaggregated peak burdens were observed in males aged 35–39 (temporal workforce engagement phase) versus females aged 55–69 (peri−/post-menopausal transition window) (Figure 6D). By 2021, this fundamental pattern persisted with reinforced divergence: male DALYs remained concentrated in younger adults (18–49 years), while female burdens dominated octogenarian/nonagenarian strata (≥80 years), particularly intensifying in nonagenarian females (≥90 years). Notably, burden peaks shifted toward older demographics—males’ zenith migrated to 50–54 years (delayed workforce attrition phase) and females’ to 65–69 years (accelerated immunosenescence period) (Figure 7D). Globally, however, male DALY rates peaked within the 35orescen49 cohort (productivity loss epicenter), contrasting with female concentration in 55–69 age brackets (caregiving transition zone) (Supplementary Figure 2).

Collectively, pancreatitis incidence and prevalence from 1990 to 2021 demonstrated persistent age- and sex-stratified patterns: males consistently bore elevated burdens in younger (20–54 years) and middle-aged (55–64 years) cohorts, while females exhibited progressive dominance in geriatric populations (≥65 years). Mortality and DALY trajectories further revealed a chronologic expansion of pancreatitis burden in aging demographics, with older adults cohorts showing amplified severity gradients over time. These epidemiologic signatures underscore dual strategic imperatives: (1) sex-differentiated prevention frameworks targeting occupational and metabolic risks in productive-age males, and (2) gero-centric care protocols addressing multimorbidity-driven pancreatitis exacerbations in postmenopausal and senescent females. Such evidence-driven stratification provides a roadmap for restructuring clinical guidelines and public health priorities, both within China’s rapidly aging context and across global populations confronting analogous demographic transitions.

**Figure 8:**
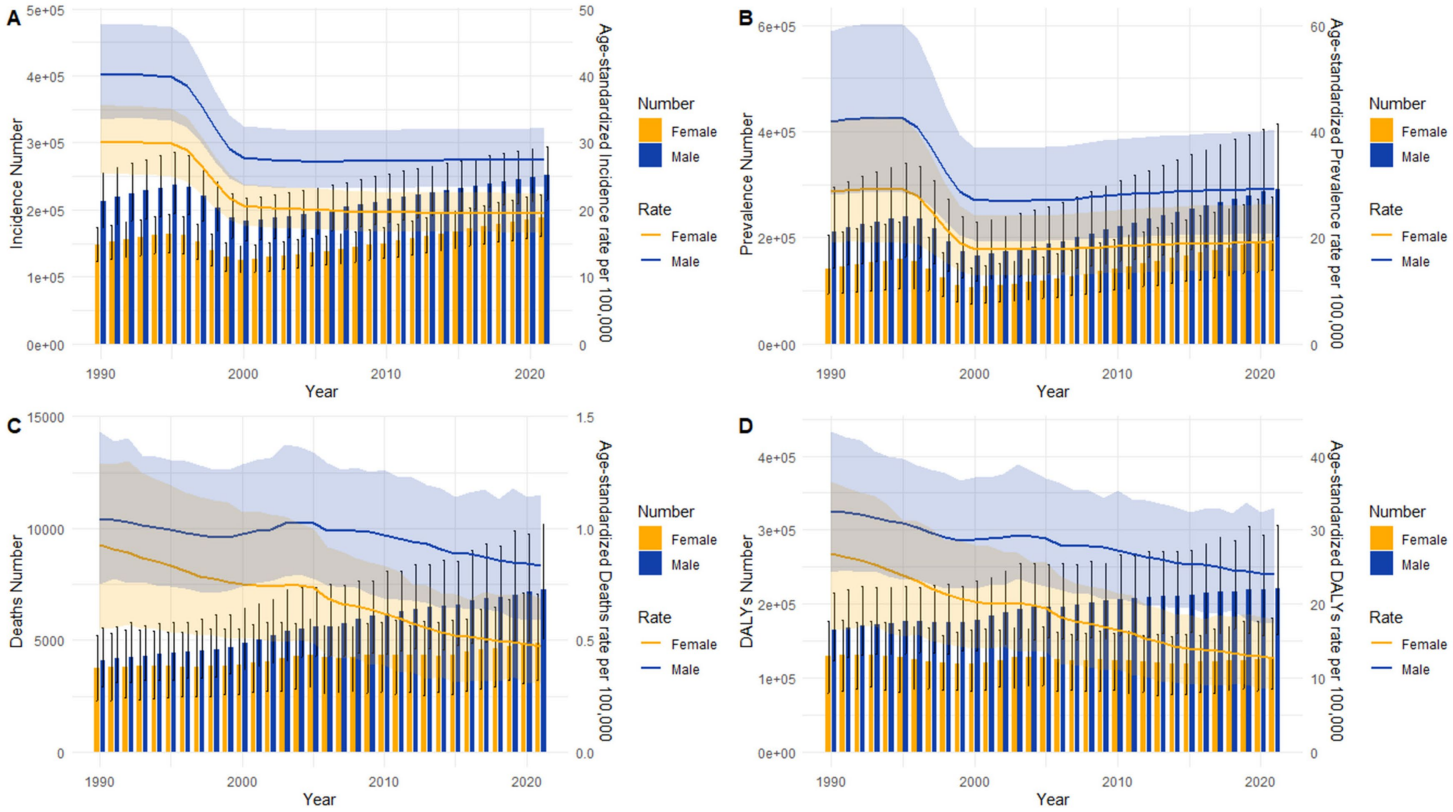
Comparisons of all-age case counts and age-standardized rates for incidence, prevalence, mortality, and DALYs between Chinese males and females, 1990–2021. **(A)** Incident case counts and ASIR (age-standardized incidence rate). **(B)** Prevalent case counts and ASPR (age-standardized prevalence rate). **(C)** Death counts and ASMR (age-standardized mortality rate). **(D)** DALY counts and ASDR (age-standardized DALY rate). Bar plots depict raw epidemiological counts; line plots represent age-standardized rates normalized to a reference population structure.

Figure 8A comparison of incident case counts and age-standardized incidence rate (ASIR) between genders (1990–2021). The incident case counts (depicted by bar plots) for both males and females have shown a steady increase over the study period, with noticeable fluctuations observed between 1990 and 2000. Throughout these years, males have consistently had higher case counts than females. In contrast, the age-standardized incidence rate (ASIR, represented by line plots) exhibited a gradual decline, accompanied by a narrowing gender disparity over time. The largest gap in ASIR between males and females occurred in 1990, but this difference significantly diminished by 2020, reflecting converging incidence trends.

Figure 8B comparison of prevalent case counts and age-standardized prevalence rate (ASPR) between genders (1990–2021). Prevalent case counts (represented by bar plots) have increased for both males and females, with male counts consistently exceeding those of females. The age-standardized prevalence rate (ASPR, depicted by line plots) exhibited a downward trend from 1990 to 2001, followed by a slight increase from 2002 to 2009, and stabilized after 2010. Notably, ASPR has consistently been lower in females than in males throughout the entire study period.

Figure 8C comparison of mortality data between genders (1990–2021). Death counts (represented by bar plots) have shown a steady increase for both males and females, with males consistently recording higher numbers than females. However, the age-standardized mortality rate (ASMR, depicted by line plots) has gradually declined over time for both genders. Notably, the gender disparity in ASMR was relatively small in 1990 but expanded to its widest gap by 2021.

Figure 8D analysis of DALYs and their age-standardized rates (1990–2021). DALY counts (represented by bar plots) have increased in males, with male counts remaining consistently higher than those of females, while female DALY counts have plateaued over time. The age-standardized DALY rate (ASDR, depicted by line plots) followed a declining trend for both genders, with trajectories similar to the ASMR patterns observed in Figure 8C.

Global trends (Supplementary Figure 3). The global age-standardized rates (ASIR, ASPR, ASMR, and ASDR) exhibited overall declines during the study period, mirroring the Chinese trends but with more moderate reductions.

## 4 Discussion

Extensive literature has documented the declining trends of pancreatitis burden globally and in China. However, many studies present these trajectories in isolation. Our study moves beyond this siloed approach by establishing a direct, head-to-head comparative framework. This framework allows us not only to confirm the declining trends but, more importantly, to quantitatively benchmark China’s progress against the global average, revealing that the pace of reduction in China has been substantially steeper. To comprehensively assess the pancreatitis burden, this study employed the Global Burden of Disease (GBD) methodology, integrating mortality, disability-adjusted life years (DALYs), age-standardized incidence rate (ASIR), and prevalence for systematic analysis across China and globally. Crucially, aging emerges as a key driver of pancreatic disease burden. The aging pancreas undergoes structural and functional degeneration (e.g., reduced exocrine capacity, fibrotic remodeling), heightening susceptibility to chronic pathologies such as pancreatitis (14). These biological correlates necessitate the development of age-specific management frameworks to mitigate disease progression and socioeconomic impacts. Furthermore, Joinpoint regression analysis was applied to scrutinize temporal trends in pancreatitis, which provided temporal granularity beyond linear trends by identifying significant inflection points. This enabled the evaluation of annual percentage changes (AAPC) between consecutive years and their statistical significance, offering potential anchors for correlating burden transitions with specific healthcare reforms over the 32-year study period.

Despite a global increase in the absolute number of pancreatitis cases from 1990 to 2021, our analysis reveals a consistent downward trend in age-standardized rates (ASRs) for incidence, mortality, and DALYs—a pattern more pronounced in China than globally. This divergence between crude and age-standardized metrics underscores the complex interplay of medical progress, demographic change, and health policy. The steeper decline in China’s age-standardized rates likely stems from synergistic advancements in clinical management and health system reforms, contextualized within its demographic transition. This accelerated decline is corroborated by an independent national burden analysis (15), and is plausibly linked to public health efforts. The effectiveness of such population-level measures is supported by global evidence, which confirms that alcohol control policies can reduce consumption and the burden of related diseases (16). In China, this is reflected in a documented reduction in the rate of frequent alcohol consumption (17). Refinements in evidence-based protocols for acute pancreatitis—including early goal-directed fluid resuscitation and step-up nutritional support—as outlined in updated national guidelines (18), have significantly improved outcomes for severe cases (19). Concurrently, enhancements in the management of chronic pancreatitis, guided by updated clinical guidelines, have reduced long-term disability (20). At the health system level, China’s healthcare reforms and the strengthening of its tiered medical care system have facilitated earlier detection and timely referral (5). This epidemiological shift is framed by demographic transition theory: while medical advancements reduce age-specific risks (captured by declining ASRs), population aging inflates the absolute number of at-risk individuals, explaining the paradox of rising crude case counts (2). However, global health inequities and disparities in regional healthcare capacity may constrain the translation of such advancements into practice. Differential access to diagnostics and therapeutics remains a critical barrier, potentially amplifying geographical divergences in disease burden trends.

Furthermore, this study highlights the significant impact of age-specific disparities on pancreatitis burden, particularly in older adults and pediatric populations. Emerging data indicate a progressive increase in pancreatitis-related burden among older adults, while pediatric cases present distinct clinical profiles and heterogeneous epidemiological patterns (21). These findings provide critical insights for developing targeted interventions tailored to specific population subgroups. Collectively, our research not only delineates temporal trends in pancreatitis burden but also offers comprehensive epidemiological evidence to inform future investigations into regional variations, risk factor prioritization, and health policy formulation. Subsequent studies should prioritize the optimization of global prevention frameworks and precision management strategies to mitigate pancreatitis burden. Emerging technologies may play a pivotal role in advancing these goals. For example, nanotechnology-based approaches—particularly those enabling targeted drug delivery and high-resolution diagnostic imaging—could refine risk stratification, enhance therapeutic specificity, and ultimately support personalized interventions tailored to disease stages or subphenotypes (22). Such innovations align with the broader objective of translating precision medicine into routine clinical practice.

The findings of this study demonstrate that although the absolute number of pancreatitis incident cases and prevalent cases has continued to rise globally and in China, age-standardized incidence rate (ASIR) and mortality rate (ASMR) exhibit a downward trend. This disparity suggests substantial advancements in global healthcare interventions and disease management over recent decades, particularly in critical care for acute pancreatitis and early diagnostic capabilities. Enhanced medical resources and the implementation of early interventions have effectively reduced pancreatitis-related lethality and disability rates, with notable progress in critical patient care significantly mitigating mortality risks (23).

Furthermore, while our national-level analysis delineates China’s overall progress against the global backdrop, it inevitably masks significant subnational heterogeneities. This study is limited by its national-level perspective, which precludes a direct examination of urban–rural and regional disparities within China. However, compelling evidence from national studies on healthcare resource allocation strongly suggests that such gradients are highly probable. Empirical research has consistently documented a pronounced ‘high-in-the-east and low-in-the-west’ geographical pattern in the distribution of healthcare resources across China, with spatial Gini coefficients for key indicators exceeding 0.5, indicating substantial inequality (24). This east–west gradient is evident at both hospital and primary care levels. For instance, the fairness of hospital resource allocation follows a pattern of western < eastern < central regions, while for primary institutions, it is western < central < eastern (25). Crucially, the distribution of highly specialized human resources—such as physicians and nurses, which are critical for managing complex conditions like severe acute pancreatitis—is particularly skewed. Studies on primary healthcare resources reveal that the ratio of medical personnel to population size is adequate in the east but falls short in the central and western regions (26). The clinical course of pancreatitis, particularly severe acute pancreatitis, is critically dependent on timely access to specialized diagnostics (e.g., endoscopic retrograde cholangiopancreatography) and advanced critical care facilities—resources that are known to be concentrated in eastern urban centers (27). Therefore, it is a logical and empirically supported inference that disparities in the availability of these specialized resources would translate into disparities in pancreatitis outcomes, such as mortality and disability rates. Consequently, our reported national averages likely represent a composite of markedly divergent local realities, underscoring an urgent need for region-specific health policies and targeted resource allocation to under-resourced regions to ensure equitable pancreatitis care outcomes nationwide.

While these trends align with reports from the Global Burden of Disease (GBD) and other studies, discrepancies may arise from variations in regional healthcare infrastructure, data sources, and technological advancements. The observed increase in pancreatitis burden across China and globally corresponds with findings from the GBD 2021 study. Similarly, Li et al. (28) and Yu et al. (3) identified rising global pancreatitis incidence, yet noted persistently escalating burdens in low-income regions due to inadequate medical resources. Our research further highlights that the growing burden of pancreatitis is closely linked to population aging, increased prevalence of chronic comorbidities, and lifestyle changes (e.g., dietary habits, rising obesity rates), a conclusion corroborated by Petrov et al. (2) and Li et al. (28).

Improvements in severe acute pancreatitis management—such as advances in endoscopic therapies, early nutritional support protocols, and critical care strategies—have markedly enhanced survival rates and reduced both mortality and disability-adjusted life years (DALYs). Additionally, advancements in healthcare systems, particularly in developed nations, have alleviated long-term sequelae through timely diagnosis and intervention (28). In China, the significant decline in ASMR is likely attributable to optimized critical-care management strategies, including early goal-directed fluid resuscitation and stepwise nutritional support, which mitigate complication risks in acute pancreatitis patients (19). However, this progress remains uneven across low- and middle-income countries (LMICs), where limited access to advanced diagnostics and therapies perpetuates high mortality rates, especially in severe acute cases. Han et al. (29) emphasize that persistent health inequities and resource scarcity in LMICs constitute major barriers to reducing pancreatitis-related mortality.

Nevertheless, despite continuous advancements in therapeutic approaches, the burden of pancreatitis remains substantial, particularly against the backdrop of a globally aging population, where the burden among older adults individuals is progressively escalating (30). This underscores that despite improved treatment protocols, significant challenges persist in managing pancreatitis within the geriatric patient population. Older adults patients frequently exhibit multimorbidity profiles—including hypertension, diabetes mellitus, and cardiovascular diseases—a complex clinical context that complicates both diagnosis and therapeutic management while heightening treatment resistance (31).

Petrov and Yadav (2) have also highlighted that insulin resistance in older adults patients may exacerbate pancreatic microcirculatory disturbances, thereby delaying inflammatory resolution. This evidence underscores the critical role of comorbidity management—such as stringent glycemic control in diabetes—as a cornerstone of comprehensive geriatric care for pancreatitis. Furthermore, aging populations face heightened burdens of pancreatitis due to age-related immune senescence and metabolic dysregulation, both of which contribute to the aggravated disease burden. These factors collectively undermine therapeutic efficacy in older adults patients, resulting in poorer clinical outcomes and increased disease severity. Notably, pancreatitis manifests distinct clinical features across age groups. In contrast to adults, the primary etiological drivers of pancreatitis in pediatric populations include infections (e.g., mumps virus), adverse drug reactions (e.g., valproic acid), and genetic predisposition (e.g., *PRSS1* gene mutations) (21).

For instance, certain genetic variants may heighten pediatric susceptibility to pancreatitis, and therapeutic regimens for this population often necessitate personalized adjustments aligned with developmental physiology (32). Therefore, early identification and targeted therapeutic interventions—such as minimizing invasive procedures and prioritizing nutritional support—are critical for mitigating the burden of pancreatitis in children (33). While geriatric populations face escalating pancreatitis burdens, pediatric cases demonstrate greater etiological complexity. This observation aligns with findings by Yu et al. (3) and Liu et al. (21), who also reported heightened pancreatitis risks in older adults patients amid advancing global aging. In older adults patients, comorbid chronic diseases and age-related immune dysfunction pose greater therapeutic challenges and increase susceptibility to complications (34). For pediatric populations, although pancreatitis incidence is relatively low, its pathogenesis is primarily mediated by infectious agents (e.g., viral infections), drug hypersensitivity (e.g., anticonvulsant reactions), and genetic predisposition—a pattern corroborated by Liu et al. (21).

The study further underscores the profound impact of geographical disparities on pancreatitis outcomes. Heterogeneity in healthcare resources, socioeconomic conditions, and health policy frameworks across nations and regions may significantly influence the pancreatitis burden. In high-income countries, robust healthcare infrastructure and integrated early diagnostic systems typically correlate with reduced morbidity and mortality rates. Conversely, low- and middle-income countries (LMICs) face persistently elevated mortality rates due to resource-constrained settings and suboptimal disease management protocols, particularly in managing severe acute pancreatitis (9, 19). These disparities are notably evident in China, where urban–rural healthcare gaps and geographical imbalances in medical resource allocation remain key drivers of differential pancreatitis-related mortality (35).

These findings collectively indicate that disparities in pancreatitis burden are primarily driven by inequitable healthcare resource allocation. In high-income countries, proactive early diagnosis and specialized critical care infrastructure have achieved significant reductions in mortality, whereas resource-limited settings face delayed clinical management due to systemic constraints (36). For example, studies by Wen et al. (4) and Jiang et al. (36) demonstrate that comprehensive healthcare systems and targeted public health interventions in affluent nations correlate with markedly lower pancreatitis-related mortality and complication rates, in stark contrast to the escalating disease burden observed in developing economies. Our study further reveals a gradually rising pancreatitis burden in China, where despite recent improvements in healthcare capacity, persistent urban–rural care disparities and regionally fragmented resource distribution remain a key contributing factor to unequal mortality outcomes (29). This evidence underscores the urgent need for globally coordinated strategies prioritizing equitable resource distribution and evidence-based health policy reform. While this study provides a comprehensive historical benchmark, the future trajectory of the pancreatitis burden remains a critical concern for healthcare planning. Building directly upon the trends quantified herein—particularly the accelerated decline in China against a slowly improving global backdrop—future research must prioritize the development of robust forecasting models. Utilizing statistical approaches such as age-period-cohort modeling or time-series analysis to project incidence, mortality, and DALYs would offer invaluable insights for anticipating healthcare needs and optimizing resource allocation. The historical patterns established in this analysis serve as the essential foundation for validating and refining such predictive efforts.

Collectively, the findings of this study align with previous research, particularly regarding the upward trajectory of pancreatitis burden observed globally and in China. Despite the persistent rise in global pancreatitis burden, evidence confirms that timely therapeutic interventions, early diagnostic protocols, and optimized healthcare resource utilization are pivotal to reducing pancreatitis-related mortality and disability. With advancements in global healthcare standards, especially in high-income nations, age-standardized mortality and disability rates associated with pancreatitis have shown significant reductions (19, 23). However, structural inequities in healthcare resource allocation persist worldwide, with acute shortages in low- and middle-income countries (LMICs) critically undermining mortality reduction efforts (37). To address this gap, context-adaptable strategies that couple cost-effective innovation with equity-driven design are urgently needed. Emerging approaches such as Just-in-Time Adaptive Interventions (JITAI)—leveraging mobile health monitoring and dynamic resource prioritization to optimize pancreatitis care in real-time (e.g., early detection of exacerbations, tailored patient triaging)—may offer a scalable model for LMICs (38). By aligning technological advancements with locally feasible infrastructure, JITAI frameworks could mitigate disparities in pancreatitis outcomes, provided systemic biases in resource distribution are concurrently addressed.

The study’s pivotal contribution resides in establishing a direct, quantitative benchmark of China’s pancreatitis burden against the world, moving beyond siloed descriptions. This comparative approach enabled a granular analysis of geographical and population-level disparities, coupled with mechanistic insights into their socioeconomic and systemic drivers that are contextualized within China’s unique trajectory. By contextualizing these findings within China’s ongoing healthcare reforms and public health initiatives, the study elucidates the multi-layered determinants of observed epidemiological shifts. Nevertheless, despite global progress in pancreatitis management, persistent challenges—including spatial resource mismatch, aging populations, and escalating comorbid chronic disease burdens—demand urgent attention. Thus, future research must prioritize three axes: (1) targeted mitigation of health inequities; (2) personalized therapeutic approaches for geriatric patients; and (3) equity-driven globalization of healthcare resource frameworks. Strategic efforts in these domains are imperative to achieving sustainable reductions in the global pancreatitis burden.

This study reveals significant shifts in the global and Chinese pancreatitis burden between 1990 and 2021, particularly in trends of incidence and mortality rates. These findings carry critically important theoretical and practical implications for optimizing public health policies and healthcare resource allocation. The data demonstrate that while the global burden of pancreatitis has increased, improvements in early diagnosis and evidence-based critical care management have driven marked reductions in mortality (2). Furthermore, personalized therapeutic strategies tailored to specific demographic groups—particularly geriatric and pediatric populations—are proven to mitigate disease burden effectively (3). By quantifying geospatial and socioeconomic disparities, this research provides robust empirical support for addressing global health inequalities and underscores the urgency of scaling up context-specific healthcare resources in low-income nations (28).

Notwithstanding these contributions, our findings must be interpreted in the context of several limitations. First, as the GBD estimates rely on modeled data and predictive algorithms, they are subject to inherent uncertainty, particularly in regions with limited primary surveillance (e.g., low-income countries), which may introduce indirect estimation biases. Second, the scope of our analysis was constrained by the available GBD metrics; clinically relevant long-term outcomes such as pancreatitis recurrence rates were not included, which limits a more comprehensive assessment of the disease’s clinical progression and burden. Third, this study is a retrospective analysis focused on historical trends and does not include forecasting models to predict the future burden of pancreatitis. Fourth, and specific to the Chinese context, our national-level analysis inevitably masks subnational heterogeneity. The GBD 2021 public database does not provide subnational data for China (e.g., provincial-level estimates), which precluded a direct analysis of urban–rural and regional (east-central-west) disparities in pancreatitis burden. Although we have cited independent studies confirming significant geographical inequalities in healthcare resources across China, future studies with access to granular, subnational data are warranted to explore this critical dimension. Despite these constraints, the GBD framework remains the gold standard for comparative epidemiological studies, and our analysis provides valuable population-level insights that lay the groundwork for future research incorporating primary, longitudinal data to elucidate finer-grained clinical dynamics.

## 5 Conclusion

Employing the Global Burden of Disease (GBD) methodology, this study systematically evaluated the pancreatitis burden across global and Chinese populations from 1990 to 2021, integrating metrics such as incidence, mortality, and disability-adjusted life years (DALYs). The analysis captures spatiotemporal patterns and highlights age- and sex-specific disparities critical for tailoring public health interventions. Key findings reveal that, despite an increase in global pancreatitis case counts (both incident and prevalent), there has been a concurrent decline in age-standardized incidence and mortality rates. These trends suggest that advancements in therapeutic protocols and healthcare infrastructure have contributed to improved outcomes. However, persistent inequities in healthcare access—particularly in low- and middle-income countries (LMICs)—and geographic disparities in disease burden among older adults populations remain significant challenges. The theoretical and practical contributions of this study provide an empirical basis for evidence-based public health strategies that integrate precision medicine with equitable resource allocation. This underscores the need for targeted therapeutic approaches for high-risk groups, including older adults individuals and populations in resource-limited settings. Emerging technologies, such as nanotechnology for targeted diagnostics and Just-in-Time Adaptive Interventions (JITAI) for real-time resource optimization, present promising opportunities to address these disparities. To translate these findings into actionable policies, a staged approach is recommended. In the short term, priorities implementable within existing healthcare frameworks include: (1) launching region-specific epidemiological studies to identify and prioritize local risk factors (e.g., biliary diseases, alcohol consumption, hyperlipidemia) for targeted primary prevention; and (2) developing and validating forecasting models using the historical benchmarks from this study to anticipate future healthcare needs and optimize resource allocation. For long-term, transformative impact, exploratory efforts should focus on: advancing the development of robust forecasting models for pancreatitis burden, building upon the historical trends established herein and evaluating the real-world effectiveness and scalability of emerging technologies, such as nanotechnology for targeted drug delivery and diagnostics, and Just-in-Time Adaptive Interventions (JITAI) for dynamic, personalized disease management in both community and clinical settings.

## Data Availability

Publicly available datasets were analyzed in this study. This data can be found at: the raw data used in this study are publicly available through the Global Health Data Exchange (GHDx) repository. A direct link to the GBD 2021 dataset platform is: http://ghdx.healthdata.org/gbd-2021.
