# Supplementary material for: Comparative analysis of trends in the burden of pancreatitis in China and worldwide, 1990–2021
Source: Front Public Health. 2025 Dec 5;13:1616215. doi: 10.3389/fpubh.2025.1616215 (PMC12714637; doi:10.3389/fpubh.2025.1616215)
Supplement: Supplementary file 1 [file Table_1.docx]

Supplementary Material

# Supplementary **Figure 1.**

****Global burden of pancreatitis by age group (1990 vs. 2021): Absolute counts and crude rates of incidence, prevalence, mortality, and disability-adjusted life years (DALYs)****

(A-D) Comparison of incidence, prevalence, mortality and DALYs by age group.


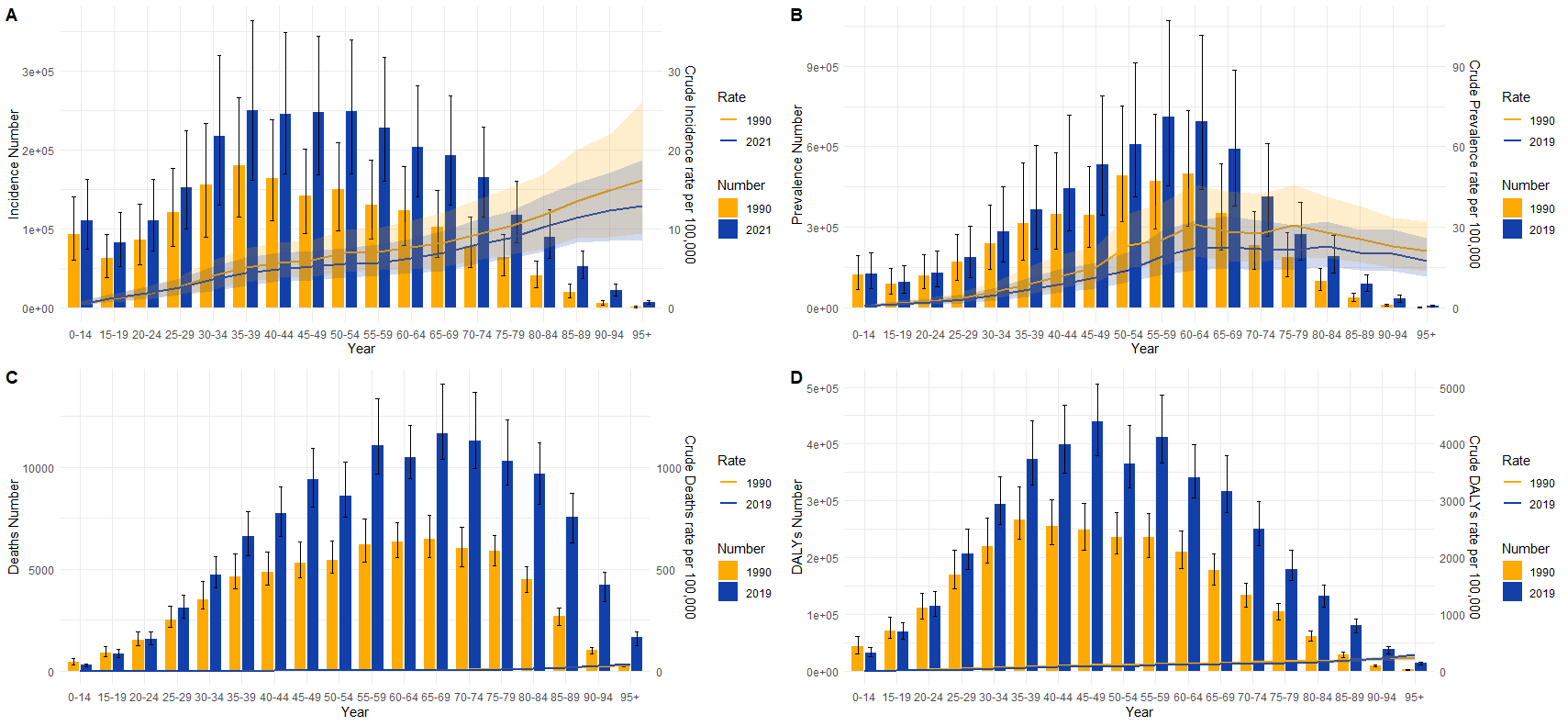


## 2 Supplementary Figure 2.

****Sex-specific burden of pancreatitis across age groups globally: Incident cases, prevalent cases, deaths, and DALYs (1990 vs. 2021)****

(A-D) Comparison of the number of incidences, illnesses, deaths and DALYs between men and women in 1990.

(E-H) Comparison of the number of incidences, illnesses, deaths and DALYs between men and women in 2021.


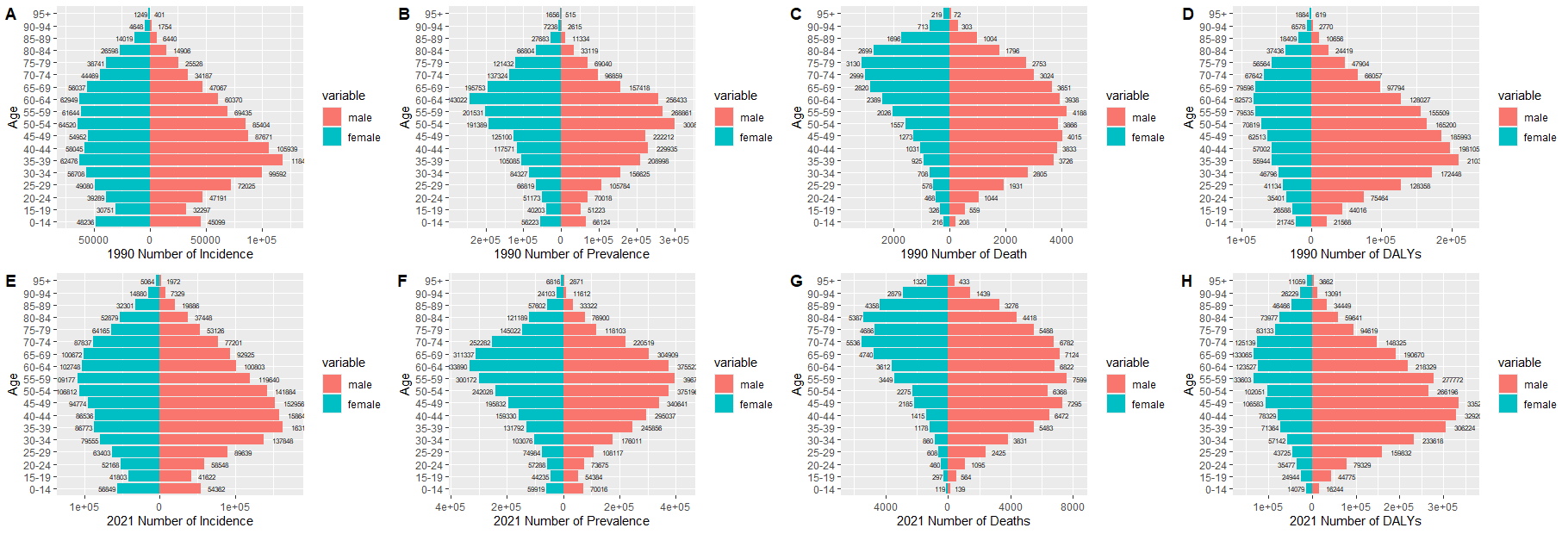


**3 Supplementary Figure 3.**

****Temporal trends in the global pancreatitis burden (1990–2021): All-age case numbers and age-standardized rates by sex****

(A-D) Age-wide numbers and age-standardized rates of incidence, prevalence, death and DALYs from 1990 to 2021.


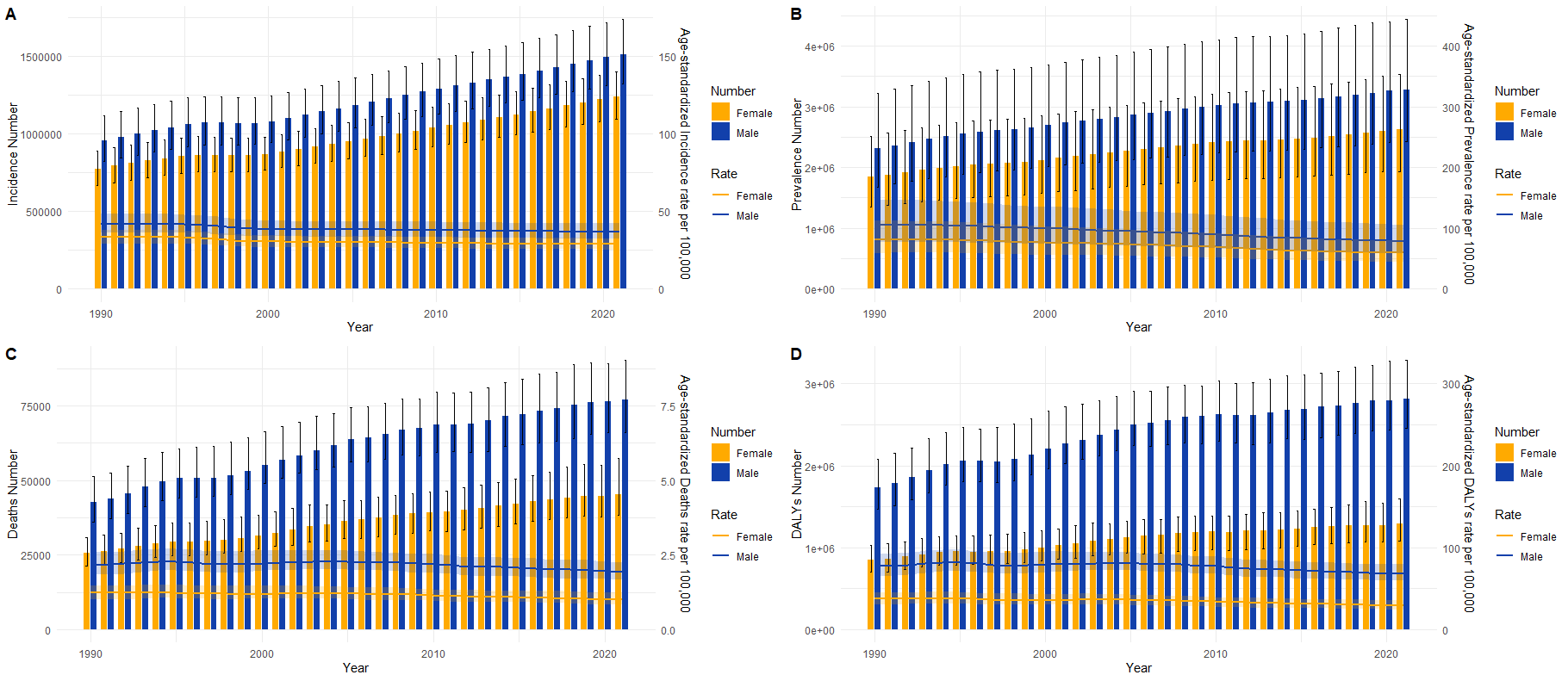


**
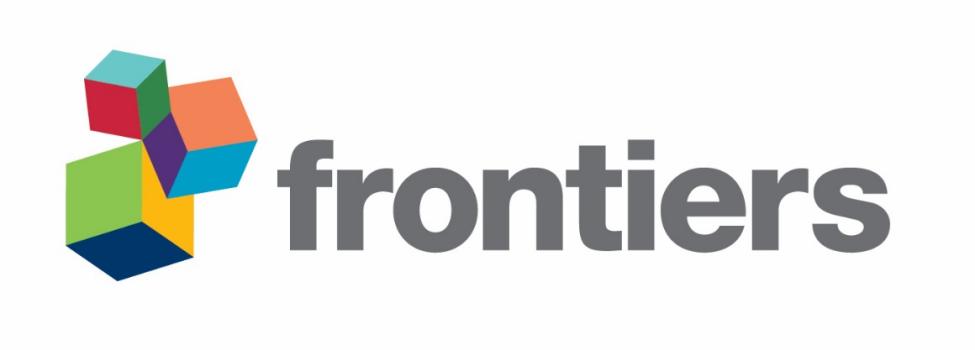
**
